# Reconstruction of Below Knee Stump with Free Plantar Fillet Flap: A Case Report

**DOI:** 10.29252/wjps.9.3.339

**Published:** 2020-09

**Authors:** Mehmet Dadaci, Mehmet Emin Cem Yildirim, Ilker Uyar, Bilsev Ince

**Affiliations:** 1 Necmettin Erbakan University, Meram Faculty of Medicine, Department of Plastic, Reconstructive and Aesthetic Surgery, Konya, Turkey;; 2 Bilecik State Hospital, Plastic Reconstructive and Aesthetic Surgery Clinic, Bilecik, Turkey;; 3 Tokat State Hospital, Plastic Reconstructive and Aesthetic Surgery Clinic, Tokat, Turkey

**Keywords:** Reconstruction, Knee stump, Free plantar fillet flap, Turkey

## Abstract

Here, we present a 45-year-old male patient who had right leg fracture in several pieces, arterial ischemia, multiple muscle, tendon losses and degloving injury on the distal thigh and knee undergoing emergency surgery due to a high-energy traffic accident and explain our experience with reconstruction of below knee stump using free plantar fillet flap in order to prevent above knee amputation in a patient with vascular injuries, multi-part fractures and soft tissue losses in the lower extremity.

## INTRODUCTION

Replantation or revascularization is the most important treatment modality in traumatic amputation and absence of circulation of the lower extremity. Although maintaining the vitality of the extremity is the primary goal, success rate of revascularization or replantation may not be high in severe crush injuries. Moreover, multiple operations, length of hospital stay, immobilization can lead to serious complications that increase mortality and morbidity, and cause a final surgical amputation.^1^

As traumatic amputation negatively affects the whole life of the patient, it is an important point to perform stump repair at the most possible distal level in limb amputations in terms of rehabilitation. Fillet flaps are obtained from amputated limbs or severed body parts that cannot be replanted or salvaged, and used for reconstruction. The fillet flaps are composite axial flaps and can provide skin, muscle, fascia and bone in reconstruction. Therefore, free fillet flaps allow flap harvesting without additional donor site morbidity and have become an accepted strategy.^1^^-^^3^ In this study, we presented our experience with reconstruction of below knee stump using free plantar fillet flap in order to prevent above knee amputation in a patient with vascular injuries, multi-part fractures and soft tissue losses in the lower extremity.

## CASE REPORT

**Fig. 1:**
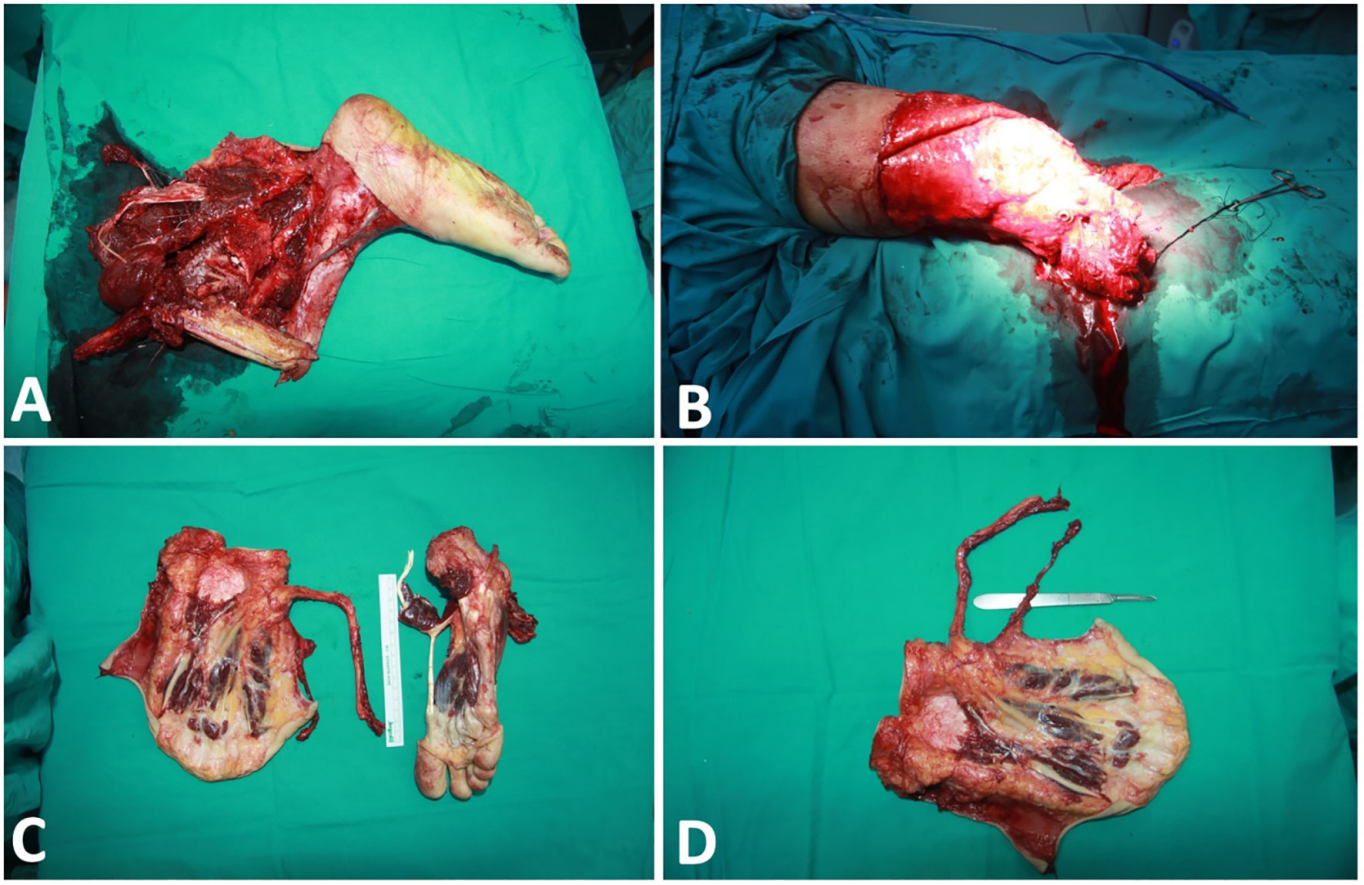
**A. **Traumatic amputated tissue. **B. **The view of the soft tissue defect of the stump. **C. **The view of the flap and donor area (right foot) **D. **The view of the fillet flap after harvesting

A 45-year-old male patient who had right leg fracture in several pieces, arterial ischemia, multiple muscle, tendon losses and degloving injury on the distal thigh and knee underwent emergency surgery due to a high-energy traffic accident (Figure 1). Transtibial amputation was performed at the level of the tibial metaphyseal region, and open reduction-internal fixation was performed due to tibial lateral plateau fracture. There was not enough soft tissue for stump reconstruction. It was observed that there was no injury in the plantar region of the amputated tissue, and the plantar fillet flap (myocutaneous free flap) was planned to be used for reconstruction of below knee stump. 

We began the operation as two teams. One team explored the arterial tibialis posterior, vena tibialis posterior and nervus ischiadicus at the recipient site, making them suitable for microsurgery. The other team simultaneously harvested the plantar fillet flap from the amputated tissue, following explorations of arterial tibialis posterior, nervus tibialis and vena saphena magna. After artery and vein anastomoses, perfusion of the flap was provided, and then nerve coaptation was performed. 

**Fig. 2:**
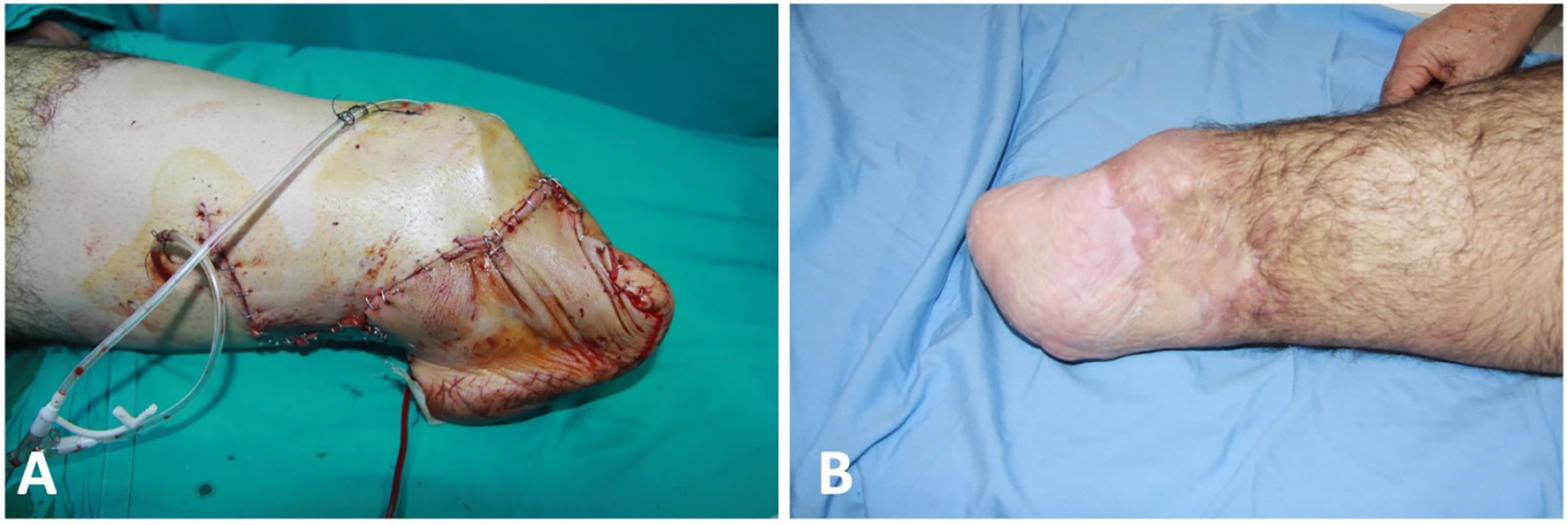
**A. **Initial view of the flap after insetting** B. **Postoperative 1^st^ year

**Fig. 3:**
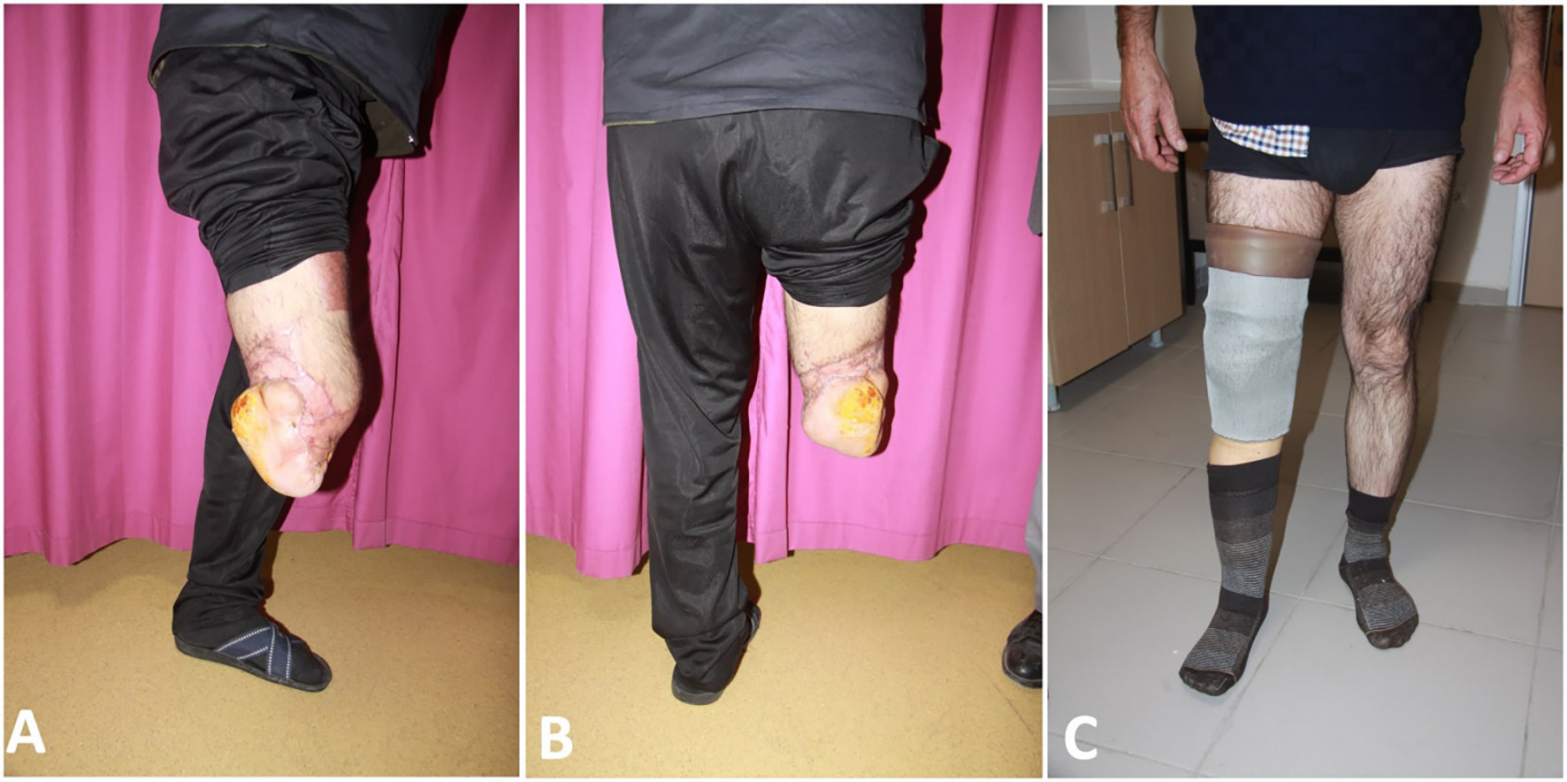
**A. **Lateral view of the reconstructed below knee stump. **B.** Posterior view of the reconstructed below knee stump. **C.** Standing position of the patient with prosthesis

The degloving injury areas of traumatic zone was excised and replaced to the defects as a full-thickness skin graft. During the follow-up period, there were no complications such as partial flap necrosis, ischemia, bleeding, hematoma, etc. However, due to necrosis of the full-thickness skin grafts at the flap superior, this area was repaired with split thickness skin graft from other thigh after debridement 2 times in 1 week intervals. The patient was discharged on the 20^th^ postoperative day (Figure 2). Physical rehabilitation of the patient was started on the 40^th^ postoperative day. Amputation was performed at the level of below knee using a free plantar fillet flap and the patient was given the chance of using prosthesis by preserving the knee joint and provided sensation of the stump (Figure 3).

## DISCUSSION

Decision on the level of amputation of an extremity is a serious challenge for a surgeon in terms of medical ethics.^4^^-^^5^ Therefore, some criteria have been reported in the literature for standardization of amputation decision. “The Mangled Extremity Severity Score” reported by Johansen *et al.* is one of them. According to this score, severity of the injury (Low – Very high energy: 1-4), limb ischemia (Pulse reduction - Cold, paralytic, 6 hours past: 1-3), shock (Systemic blood pressure ≥9 mmHg: 0 -persistent hypotension: 2) and age (under 30 years - 50 years and older: 0-2) parameters were scored. Direct amputation was recommended, if this score was 7 or more.^5^

In our case, this score was calculated as 10, and the patient had limb ischemia and hypotension exceeding 6 hours with dirty crushed injury. There are few studies in the literature in which plantar fillet flap is used as a free flap and has been reported. In 2003, Küntscher *et al.*^6^and in 1995, Goldberg *et al.*^7^ performed free plantar fillet flap to repair sacral decubitis ulcers in paraplegic patients. In addition, the outcomes of 8 patients who underwent foot fillet flap were presented by Tos *et al.*^8^ that two of 8 patients were traumatic amputations.

However, in these two cases, the calcaneus was included in the flap and the stump length was increased by fixing the calcaneus to the tibia. In both of these cases, due to the bone fixation, healing and days without weight bearing took more time (80-180 days). As well, one of them had non-union defect. In our case, as there was no bone fixation, the patient was discharged on the 20^th^ day and the rehabilitation process was started on the 40^th^ day. Except for these studies, there were no reports of free plantar fillet flap either in early or late reconstruction of traumatic amputations or in other reconstructions in the literature.

## CONCLUSION

In our case, the knee joint was preserved and given the chance of effective prosthesis application and rehabilitation. Finally, the patient was satisfied with this result and was able to overcome the psychosocial and physical difficulties in his new life. There are conscientious and ethical dimensions for surgeons to decide the amputation and to determine the amputation level. In the surgical treatment of traumatic amputations, it is very important to initiate the rehabilitation process early with appropriate prosthesis and choosing the most distal level.
